# Mediating effect of sleep disturbance and rumination on work-related burnout of nurses treating patients with coronavirus disease

**DOI:** 10.1186/s40359-022-00905-6

**Published:** 2022-08-12

**Authors:** Salman Zarei, Khadijeh Fooladvand

**Affiliations:** Psychology Department, Lorestan University, Khorramabad, Iran

**Keywords:** Burnout, Nurse, Psychological distress, Rumination, Sleep disturbance

## Abstract

**Background:**

COVID-19 has created significant and unprecedented psychological distress on nurses working with COVID-19 patients. Nurses dealing with such psychological distress are prone to burnout. This study examined the mediating role of sleep disturbance and rumination in the association between psychological distress and work-related burnout of nurses treating COVID-19 patients.

**Methods:**

A cross-sectional study was conducted from 26th February to 16th March 2021, on a sample of 250 nurses who were actively working during the COVID-19 pandemic in five referral hospitals in Tehran, Iran. The Oldenburg Burnout Inventory, Kessler Psychological Distress Scale (K10), Pittsburgh Sleep Quality Index, and Ruminative Responses Scale were used to collect data. Data analysis was based on pearson’ correlation analysis and path analysis.

**Results:**

Psychological distress has a significant effect on job burnout. When sleep disturbances were modeled as a mediator, path coefficients of psychological distress showed a significant effect on job burnout. Also, according to the findings, rumination poses a significant mediating effect on the association between psychological distress and job burnout.

**Conclusion:**

This study demonstrated the importance of designing psychological interventions intended to reduce sleep disturbances and rumination when experiencing stressful events to avoid job burnout among nurses.

## Background

As the COVID-19 rapidly turned into a pandemic there was not enough time to share epidemiological information necessary to control the disease or to design surveillance systems thus, resulting in a major burden [1]. Some studies emphasized healthcare professionals, particularly nurses, are at an increased risk of developing negative psychological consequences when faced with high-risk and stressful pandemic environments [2]. There is no doubt pandemics carry a heavy psychological burden on healthcare professionals, which is due to factors related to the workplace and individual characteristics [3]. Nurses who come in direct contact with infected individuals can experience major negative feelings like anger and frustration as well as feelings of loneliness [4, 5]. According to a systematic review and meta-analysis about one-third of nurses who directly worked with COVID-19 patients experienced psychological distress symptoms like stress, anxiety, and depression [6]. Recently published study in Iran show that among all participated nurses, 72.8% and 42.4% of the subjects reported anxiety and depression, respectively [7]. Similarly, another study in KSA revealed that the prevalence of psychological distress reported by healthcare workers especially nurses during the COVID-19 pandemic was high, ranging from mild-moderate to severe in severity [8].

Elevated levels of stress, anxiety, depression are the manifestation of psychological distress among nurses [9]. Psychological distress, fatigue, and higher workload – all contributing factors to burnout—may cause musculoskeletal problems in nurses [10, 11]. Resource allocation theory posits that negative thoughts related to psychological distress symptoms consume a certain amount of cognitive resources, leading to insufficient cognitive resources to deal with more important tasks [12, 13], which will decrease one’s work enthusiasm, self- confidence, and lead to burnout in the long run [14]. In the same vein, some studies conducted during the COVID-19 pandemic reported higher levels of moderate to severe stress, anxiety and depression, along with higher rates of burnout among nurses [9, 15]. Similarly, other cross-sectional studies found that higher levels of psychological distress increase the likelihood of more severe burnout, especially among nurses [16–18]. Burnout is a psychological syndrome resulting from prolonged exposure to job stress or physically demanding work-related conditions [19]. Previous studies have shown that work-related burnout is linked to stressful workplaces and leads to the poor health of nurses [20, 21]. In addition, studies showed that socio-demographic characteristics include gender, age, marital status, level of education and work experience are influential factors in experiencing burnout [22, 23]. Burnout is prevalent among nurses taking care of COVID-19 patients [24], and they are likely to have poor work performance and more fatigue [25].

Systematic reviews and meta-analyses show that nurses had moderate to high levels of burnout during COVID-19 [26]. A systematic review of 7 studies showed that among these articles, four (57.14%) reported moderate burnout and three articles (42.86%) reported high level of burnout among nurses during the COVID‑19 pandemic [27]. Another meta-analysis involving 49 countries revealed that the overall prevalence of burnout out was 11.23% [28]. In a study in the Uganda the results show that 40% (n = 158), 41.77% (n = 165) and 18.23% (n = 77) reported high, average and low levels of burnout, respectively [29]. Similarly, a systematic review and meta-analysis showed that the overall prevalence of nurses' burnout during the COVID-19 pandemic was 20.6% [30]. Nevertheless, the experience of psychological distress like anxiety and stress does not always result in job burnout as the development of burnout attitudes and behaviors may involve other intermediary mechanisms jointly responsible for such a relationship. Some researchers [31] indicate that psychological distress can be associated with job burnout beyond the direct links, and there is a need to explore more complex relations between both.

Recently published studies reported an association between sleep quality and burnout syndrome. Such findings (e.g., sleep quality, reduction of burnout) have been used to help nurses recover from fatigue and psychological distress caused by work [32]. Studies have shown that frontline healthcare workers are experiencing sleep loss during the current COVID-19 pandemic [33]. A social media survey study reported that nearly 96% of participants reported poor sleep. One-third (30%) reported moderate or severe insomnia. Many participants (60.9%) experienced sleep disruptions due to device use or had nightmares at least once per week (45.2%) [34]. Similarly, another study revealed that of the 987 participated frontline nurses who are in direct contact with patients with COVID-19, 58.8% (n = 580) reported poor sleep quality [35]. Either long shifts or changes to shift rotations may contribute to sleep disturbance that paves the way for job burnout [36], which is the pathological consequence of a stress-related process characterized by emotional exhaustion and disengagement [37]. It is well established that adequate sleep can reduce burnout, mainly through restoring energy [38]. In a cross-sectional study, nurses who dealt with COVID-19 cases suffered from extreme psychological distress and sleep disturbances that pave the way for burnout and decreased capacity to continue work [39]. Therefore, the researcher expects that psychological distress will predict increases in nurses’ sleep disturbances. Furthermore, the researcher expects that sleep disturbances will predict relative increases in nurses’ job burnout. Thus, it is anticipated psychological distress will predict job burnout, through sleep disturbances (Hypothesis 1).

Grounded in resource allocation theory [12], the current study proposes rumination as another possible mediator between nurses’ psychological distress and job burnout. Negative appraisal of stressful events, mainly as a threat, is at the core of the cognitive model of stress. This appraisal not only can cause psychological distress, but also lead to rumination of the experienced event, which results in stress [40]. Rumination is a method of coping with negative moods and involves a typical negative information processing bias that maintains the emotional influence of negative life events [41]. Research indicates that individuals ruminating during stressful events is one of the most important factors affecting the consequences of events, including burnout [42]. Moreover, these studies reported that rumination serves as a mediator [43]. For example, concerning the association between stress and job burnout among ICU nurses, affective rumination acts as a mediator [44]. The same is true concerning the association between anxiety and exhaustion, which is of crucial importance for job burnout in nurses [45]. Subsequently, in a study on a group of working adults, Boren [46] found a direct association between co-ruminations and burnout. Accordingly, the researcher expects psychological distress will predict increases in nurses’ rumination. Given extant research has demonstrated that rumination is a predictor of a relative rise in job burnout among nurses. It is hypothesized psychological distress will predict job burnout, through rumination (Hypothesis 2).

According to the literature, nurses suffering from job burnout not only are prone to job dissatisfaction but are also more likely to leave their jobs [47, 48]. Burnout in nurses has consistently shown a dose–response relationship (i.e., the relationship between the amount of exposure to a stressor and the resulting changes in health) with poorer patient safety outcomes [49]. In addition, marital stress, substance abuse, and suicide ideation can all be major consequences of burnout among nurses [50]. Apart from its negative impacts on health and wellbeing, job burnout is associated with increased medical errors and decreased quality of services. Hence, identifying factors that contribute to job burnout among nurses is of high value. The present study intended to provide empirical evidence regarding the association between psychological distress and nurses’ work-related burnout to expand our knowledge about the impact of psychological distress on job burnout in nurses who deal with COVID-19 patients.

## Methods

Following a cross-sectional online survey, the current study was carried out from 26th February to 16th March 2021 on all nurses who were working with COVID-19 patients in a referral hospital in Tehran, Iran. We initially recruited 275 nurses from five referral hospitals. While there is no consensus about the sample size of studies intended to perform path analysis, several researchers recommended minimum sample size of 200 participants [51].The inclusion criteria were as follow: [1] Being a licensed nurse; [2] having a minimum working experience of one year; [3] directly providing care to patients with COVID-19. The exclusion criterion was having a psychological disorders background [based on participants’ self-report]. Before completing the formal survey, informed consent was obtained from all participants. Hence, only those who agreed to participate could complete the questionnaire. In addition, participants were informed they could withdraw from the study at any time and they were ensured confidentiality of their data.

### Research instruments

Data was collected using a self-report, structured online survey with items on socio-demographic [age, gender, degree level, work experience, and marital status] as well as four psychometric measures intended to examine the study variables.

### Ruminative responses scale [RRS]

The RRS is a subscale of the Response Styles Questionnaire [RSQ] that contains 22 items scored on a four-point Likert scale, ranging from "*almost never*" [1] to "*almost always*" [4]. This scale intends to describe the response to symptoms caused due to stressful events. The RRS consists of three factors: symptom rumination, brooding, and reflective pondering [52]. The Iranian version of RRS has been widely used and has reported good reliability and validity among nurses [53]. In present research, the Persian version of the RRS demonstrated good internal consistency (Cronbach’α = 0.91).

### Pittsburgh sleep quality index [PSQI]

The PSQI contains 19 items on seven components related to sleep quality [i.e., subjective sleep quality, sleep latency, sleep duration, habitual sleep efficiency, sleep disturbances, use of sleeping medications and daytime dysfunction] intended to evaluate the sleep quality during the past month. It is scored on a four-point Likert scale, ranging from "*no difficulty*" [0] to "*severe difficulty*" [3]. The total score of PSQI ranges from zero to 21, in which a score lower than five indicates good sleep, while a score higher than five indicates poor sleep quality [54]. The PSQI has been widely used in Iranian nurses’ samples and shows great reliability and validity [55]. In this research, the Persian version of the PSQI demonstrated good internal consistency (Cronbach’α = 0.88).

### Kessler psychological distress scale [K10]

The K10 is a 10-item scale intended to measure psychological distress. It covers dimensions of nervousness, hopelessness, sadness, worthlessness, and fatigue experienced by respondents during the past month. It is scored on a five-point Likert scale, ranging from "*none of the time*" [1] to "*always* " [5]. The total score ranges from 10 to 50, with higher scores indicating more psychological distress [56]. The instrument’s reliability and validity are further evidenced by widespread use of the K10 in research on Iranian nurses [57]. In this study, the Persian version of the K10 demonstrated good internal consistency (Cronbach’α = 0.83).

### The oldenburg burnout inventory [OLBI]

The OLBI was designed to measure burnout among individuals working in various occupational groups including nursing, consisting of 16 items that cover two areas: Exhaustion and Disengagement. Each dimension has eight items scored on a four-point Likert scale, ranging from “*strongly disagree*" [1] to "*strongly agree*" [4]. It is worth noting that the higher the score, the more severe the level of exhaustion and disengagement, which translates into an increased risk of burnout [58]. The Iranian version of the OLBI has been proven to be a valid and reliable scale [59]. In the present study, the Persian version of the OBI demonstrated good internal consistency (Cronbach’α = 0.89).

### Statistical analysis

The data were analyzed using SPSS 23.0. We first ran the descriptive statistics of the socio-demographic characteristics in nurses. Then, Pearson’s correlation tests were performed to explore the associations among psychological distress, sleep disturbance, rumination and work-related burnout. Finally, we used AMOS 20.0 to evaluate our models based on the following model fit indices, chi-square (*x*^2^), comparative fit index (CFI), Tucker–Lewis index (TLI), standardized root-mean square residual (SRMR), and root-mean square error of approximation (RMSEA). We examined our model fit based on Hu and Bentler’s guidelines [60].

## Results

Fifteen participants missed more than 50% of items on a given scale and were excluded from subsequent analyses. Therefore, the effective sample comprised 250 nurses (91% response rate), with 185 female [74%] and 65 male [26%]. Participants had an average age of 37.62 years (SD = 5.13) and 81.2% (n = 203) were married. Further, 59.2% (n = 148) had a work experience of five years, and 71.2% (n = 178) had a Bachelor of Nursing as the highest level of education. Full socio-demographic characteristics of the sample are presented in Table 1. The independent sample *t*-test of OLBI scores showed no statistically significant difference according to gender (*t* = 0.19, *P* = 0.85), and marital status (*t* = 0.26, *P* = 0.71). In addition, the one-way ANOVA showed no statistically significant differences in OLBI scores in terms of degree levels (*F* = 1.15, *P* = 0.31).

**Table 1:** Socio-demographic characteristics of nurses (N = 250)

Characteristics	Numbers	Percentage
Gender		
Female	185	74%
Male	65	26%
Marital status		
Married	203	81.2%
Single	47	18.8%
Degree Levels		
Bachelor	178	71.2%
Master	56	22.4%
Doctoral	16	6.4%
Work Experience		
1–5	148	59.2%
6–10	72	28.8%
> 10	30	12%

Descriptive statistics for all questionnaire measures and the results of normality and reliability tests are reported in Table 2. The result of Kolmogorov–Smirnov test showed that the distributions of study variables are normal (*P* > 0.05).

**Table 2:** Descriptive statistics, normality and reliability tests

	Mean ( SD)	Kurtosis	Skewness	Kolmogorov–Smirnov		Cronbach’s Alpha
				Statistic	Sig	
Psychological distress	29.57 (6.03)	− 0.79	0.81	0.07	0.061	0.83
Sleep disturbance	8.93 (2.09)	1.17	− 0.63	0.05	0.082	0.88
Rumination	42.65 (7.37)	0.82	0.74	0.07	0.055	0.91
Work-related burnout	17.43 (4.55)	− 0.82	1.33	0.09	0.052	0.89

In the first step of the statistical analysis, it was decided to check whether there were any significant relationships between the tested variables. In order to select an appropriate analysis, it was checked whether the distributions of the examined variables showed large asymmetry. Skewness and kurtosis statistics showed that the studied distributions did not show large asymmetry (Table 2). On this basis, a parametric Pearson’s r correlation analysis was performed. Our analyses revealed that all our study variables were significantly correlated with each other. (*P* < 0.01). More detailed data are shown in Table 3.

**Table 3:** Correlation between for study variables and collinearity statistics

Variables	Pearson correlation coefficient				Collinearity Statistics	
	(1)	(2)	(3)	(4)	Tolerance	VIF
(1) Psychological distress	1				0.71	6.27
(2) Sleep disturbance	0.55**	1			0.62	5.91
(3) Rumination	0.61**	0.57**	1		0.56	4.33
(4) Work-related burnout	0.45**	0.52**	0.49**	1	–	–

*P* < 0.01**

In the next step, it was decided to perform a mediation analyses due to the significant relationships found using the correlation analysis. We conducted confirmatory factor analysis (CFA) to examine the goodness of fit. CFA was used to test the adequacy of our research model’s measurement before conducting an analysis of the structural model. To evaluated model fit, we used the cutoff criteria of CFI ≥ 0.95, SRMR, and RMSEA ≤ 0.08. Our research model which was comprised of 4 factors (psychological distress, sleep disturbance, rumination and job burnout) demonstrated the best model fit indices compared with other measurement model. Table 4 displays the results of the CFA of measurement model.

**Table 4:** General fit assessment indices of research model

Indices	CMIN/df	SRMR	CFI	TLI	RMSEA
Research Model	1.63	0.05	0.96	0.96	0.06
Status	Good	Good	Good	Good	Good

n = 250, CFI = comparative fit index, TLI = Tucker-Lewis index, RMSEA = root-mean square error of approximation,SRMR = standardized root-mean square residual

Next, we tested a structural model to examine our hypotheses. The model consisted of one exogenous factor (psychological distress) and three endogenous variables (sleep disturbance, rumination and job burnout). Our hypothesized structural model also demonstrated a good fit to our data: CMIN/df = 1.63; CFI = 0.96; TLI = 0.96; SRMR = 0.05; RMSEA = 0.06; 90% confidence interval (CI) [0.04, 0.10].

**Fig. 1:**
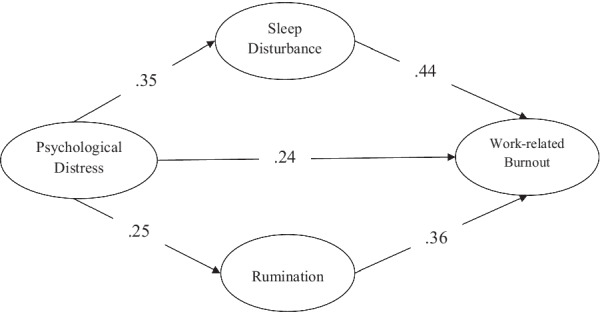
Final mediation model with standardized path coefficients

Figure 1 shows the result indicating that psychological distress had a statistically significant direct effect on sleep disturbance [β = 0.35, C.R = 6.78, *P* = 0.000], on rumination [β = 0.25, C.R = 5.38, *P* = 0.010] and on work-related burnout [β = 0.24, C.R = 5.17, *P* = 0.011]. In addition, the direct path from sleep disturbance to work-related burnout [β = 0.44, C.R = 7.99, *P* = 0.000] and the direct path from rumination [β = 0.36, C.R = 6.01, *P* = 0.000] to work-related burnout was statistically significant.

In the next step, as shown in Table 5, we examined the hypothesized indirect path by using 5000 bootstrapping samples and bias-corrected confidence intervals. The results revealed that psychological distress had statistically significant indirect effects on work-related burnout (standardized indirect effect = 0.15, *P* = 0.004, BC 95% CI [0.06, 0.18]) via sleep disturbance. In addition, the results revealed that psychological distress had statistically significant indirect effects on work-related burnout (standardized indirect effect = 0.09, *P* = 0.009, BC 95% CI [0.02, 0.11]) via rumination. To validate our hypothesis, we employed bootstrapping using a 95% confidence interval, and the result was that no confidence intervals included zero. These reveal the effects of psychological distress on work-related burnout mediated by sleep disturbance and rumination.

**Table 5:** Standardized indirect path and bootstrapping test

Indirect path	Bootstrapping		BC 95% CI	
	Indirect effect	SE	Lower Bound	Upper Bound
Psychological distress → Sleep disturbance → Work-related burnout	0.15	0.013	0.06	0.27
Psychological distress → Rumination → Work-related burnout	0.09	0.010	0.02	0.11

## Discussion

This study examined the mediating effect of sleep disturbance and rumination in the association between psychological distress and work-related burnout in nurses treating patients with COVID-19. The results showed that psychological distress through sleep disturbance exerts an indirect effect on work-related burnout in nurses, revealing lower levels of sleep quality among nurses who suffer from higher levels of stress and anxiety as well as the increased risk of burnout (hypothesis 1). This finding aligns with previous findings that demonstrated sleep disturbance serves as a mediator [32, 39]. It can be attributed to the fact that sleep plays a unique role in restoring daily functioning and regulating emotional feelings, which in turn can act as a mediating factor for the association between stress and negative effects of burnout and help the brain process emotionally stressful events in adaptive ways [38].

Furthermore, it can be attributed to the association between low sleep quality and the inability to unwind or disengage from thoughts of work during leisure time, which its role in predicting burnout is well established [36]. Low sleep quality not only has negative consequences, including impaired emotional functions, but also causes emotional instability, higher irritability, and short-temperedness, which in turn may result in exhaustion that has a unique role in the development of job burnout [61]. The negative effect of psychological distress on normal sleep patterns of nurses, which paves the way for the development of burnout, may be another important explanation [14]. Also, sleep deprivation poses considerable negative effects on prefrontal cortical functioning. Improving prefrontal cortical functioning stimulates behavioral responses, based on cognitive and social context can cause increased sensitivity to stressful stimuli and events that may result in an increased risk of disengagement from work, therefore leading to job burnout [62].

In the present study, rumination presented a mediating effect on the association between psychological distress and work-related burnout in nurses, indicating that nurses with higher levels of psychological distress tend to ruminate more and there is an enhanced risk of experiencing burnout [hypothesis 2], which aligns with the results of previous studies. Particularly in that resource allocation theory demonstrated rumination serves as a mediator [43–45]. A possible explanation might be depletion of cognitive resources in the face of stressful events, because ruminative thoughts, related to negative emotions irrelevant to the tasks, consume limited available resources, which results in poor functions in the short term and burnout in the long term [40]. According to the literature, repetitive and intrusive thoughts are the main attributes of rumination. In addition, there are evidence regarding the positive association between rumination and depression, stress and anxiety [63]. Confronted with negative events, nurses may lapse into brooding about their impact and the associated negative emotions [42]. Hence, it is reasonable to assume nurses with higher levels of psychological distress and negative emotions during the pandemic are more likely to use ruminative coping techniques. While nurses with a high level of rumination are very likely to experience work-related burnout. On the other hand, those with a low level of rumination can effectively control their thinking logic during negative events, which indicates their higher ability to manage negative experiences [64]. As a result, they can better reduce feelings of hopelessness as well as negative emotions, which is highly useful to reduce the negative consequences of burnout.

A few limitations should be taken into consideration when interpreting findings. Firstly, the cross-sectional design is not appropriate for the evaluation of causal relations, which probably has affected the validity of the findings. Hence, further studies with either longitudinal or experimental designs are required to expand our knowledge about this association. Secondly, data was collected through self-report measures, which it might have affected the validity of the findings. Future studies should use multiple methods (e.g., standardized interview and observation) to collect data, which not only will it provide more detailed evidence, but also can reduce potential respondent biases. Lastly, all participants were working in public referral COVID-19 hospitals in Tehran, Iran. Hence, caution should be taken when generalizing the findings. Limitations aside, the current study provided some theoretical and clinical implications. The findings can be applied to develop targeted preventive interventions focused on job burnout in nurses. Hence, increasing the awareness of healthcare professionals, particularly those working in the field of mental health, about the potential negative effects of poor sleep quality is of paramount importance to address this issue. Notably by increasing the awareness of healthcare professionals, including nurses, regarding the cognitive strategies, they can better cope with negative events that cause burnout, mainly through negative cognitive strategies and develop positive cognitive strategies. Moreover, hospitals should also provide more psychological counseling and group counseling services to nurses to help them to cope with sleep disturbances and to better manage their rumination.

## Conclusion

The current research tested a mediation model to examine the sleep disturbance and rumination underlying the relationship between psychological distress and job burnout among Iranian nurses treating patients with coronavirus disease. In brief, according to the findings, psychological distress is a risk factor for poor quality sleep and rumination, and it predicted work-related burnout through sleep disturbance and rumination. These findings substantially contribute to our understanding of psychological distress symptoms and job burnout. This likelihood demonstrated the superposition effects of these risk factors. This study is innovative in exploring sleep disturbance and rumination as mediator variables that are not considered by previous studies. Particularly in the COVID-19 epidemic today, more attention should be given to this constructs.

## Data Availability

The dataset used and analyzed during the current study are available from the corresponding author on reasonable request.
